# Reproducibility of radioactive iodine uptake (RAIU) measurements

**DOI:** 10.1002/acm2.12217

**Published:** 2017-11-22

**Authors:** Matthieu Pelletier‐Galarneau, Patrick Martineau, Ran Klein, Matthew Henderson, Lionel S. Zuckier

**Affiliations:** ^1^ Division of Nuclear Medicine The Ottawa Hospital Ottawa Ontario Canada; ^2^ Children's Hospital of Eastern Ontario Ottawa Ontario Canada

**Keywords:** hyperthyroidism, radioiodine therapy, radioiodine thyroid uptake, reproducibility

## Abstract

**Background:**

Measurement of radioactive iodine uptake (RAIU) is an important aspect of the assessment and treatment of patients with hyperthyroidism. Its uncertainty affects how much of a true change in RAIU can be detected as well as appropriateness of the therapy dosage upon which it is based. In this study, a method of estimating the reproducibility and least significant change (LSC) values for RAIU measurements, and the implications of the values observed are discussed, with emphasis on application to quality assurance initiatives.

**Methods:**

We prospectively studied 36 consecutive patients referred for RAIU measurements. Twenty‐four hours after oral administration of 370 kBq of ^131^I‐NaI in capsule form, RAIU measurements were obtained in duplicate using a thyroid probe uptake system. Assessment of reproducibility was performed using root‐mean‐square standard deviation.

**Results:**

Average difference between duplicated RAIU measurements in our study cohort was −0.1 ± 1.6% and ranged from −4.8% to 3.1%. Reproducibility of probe‐based RAIU measurement was calculated to be 1.1% and 95% LSC was 3.2%.

**Conclusion:**

In our clinic, probe‐based RAIU is a reproducible and relatively precise measurement. Using the method we have outlined, each institution can perform reproducibility assessment and compute the LSC of RAIU measurements based on its own staff, iodine isotope, equipment, protocols, and patient population. These values are useful in the assessment of serial change in RAIU, and as more experience is accumulated, can serve as benchmarks to be used in quality assurance initiatives.

## 1 INTRODUCTION

Quantification of radioactive iodine (i.e., iodine‐131) uptake (RAIU) is a standard, widely‐accepted method of estimating thyroid hormonogenesis based on the degree of trapping and organification of radioactive iodine in the thyroid gland. The measurement of RAIU was introduced into clinical practice over 60 yr ago^1^, ^2^ and currently remains an important tool in the investigation of various etiologies of hyperthyroidism.^3^, ^4^, ^5^, ^6^, ^7^, ^8^ Twenty‐four hour (24‐h) RAIU is also a key component of the formula used to calculate the dosage of iodine needed when treating hyperthyroidism with radioactive iodine therapy (RAIT).^9^, ^10^, ^11^, ^12^, ^13^ Based on a prescribed activity concentration in the thyroid at 24 h post administration (K, in units of MBq/g) and the fractional 24‐h RAIU, the therapeutic iodine activity is given by the formula below, with K typically ranging from 3.0 to 8.1 MBq/g:^3^, ^14^, ^15^, ^16^

(1) Dose (MBq)=K(MBq/g)×thyroid weight (g)/24‐hRAIU

Very important requirements in medical testing are, of course, reproducibility and accuracy of measurements. For any clinically useful test, the variance of measurement should be much smaller than the actual variability in the characteristic or parameter measured. To our knowledge, however, reproducibility of RAIU measurements has not previously been investigated. This is in contrast to bone mineral densitometry, for example, where consideration of reproducibility is central to its utility in clinical decision making.^17^, ^18^ The purpose of the current study, therefore, was to develop a method of estimating clinical reproducibility and least significant change (LSC) values for RAIU measurement. Reproducibility of measurement is important in the analysis of sequential changes in RAIU over time and in quality assurance of the measurement. The methods we describe can be adopted by other laboratories to estimate this parameter in their own practice. Measurement of reproducibility, especially in comparison to historical and peer‐based values, can be incorporated into quality assurance programs.

## 2 MATERIALS AND METHODS

The study was conducted under the approval of the Ottawa Hospital Research Ethics Board. To estimate reproducibility of measurement, we analyzed 36 consecutive patients referred for thyroid scintigraphy and RAIU at our two affiliated sites, which employ identical thyroid uptake systems, each consisting of a 2″ NaI crystal, photomultiplier tube, and multichannel analyzer (Capintec Thyroid Uptake System, Captus© 3000). RAIU is measured 24 h after oral administration of approximately 370 kBq of ^131^I‐NaI in capsule form (Jubilant‐DraxImage, Kirkland, Quebec) following standard procedure guidelines.^19^ Briefly, prior to administration, the capsule of ^131^I‐NaI is placed in a neck phantom holder and counts are measured using the gamma probe; a background count without the capsule is also obtained. At time of uptake, typically 24 h following administration, similar measurements are performed over the patient's neck and thigh (patient background), using identical 1‐min acquisitions and a 364 keV ± 10% photopeak energy window. RAIU is computed as the ratio of background‐corrected 24‐h neck counts to background‐corrected capsule counts; as noted, neck background is approximated by count rate over the patient's thigh.

For the purpose of obtaining reproducibility estimates, we varied our standard protocol only in that each measurement (capsule, background, 24‐h neck, 24‐h thigh) was performed in duplicate. Repositioning of patients and capsules was also performed between duplicate measurements to simulate variability in the uncertainty estimation due to positioning. Final diagnosis of patients is based on available clinical information, blood work, and thyroid scintigraphy as well as RAIU.

### 2.A Statistical analysis

Repeated measurements were compared using a Bland–Altman analysis. The difference between first and second measurements was assessed with a Student's paired t‐test. A Pearson product moment correlation coefficient was calculated to assess the correlation between difference (ΔRAIU) and average of the duplicated RAIU measurements. Based on the set of 36 duplicate measurements, empirical assessment of reproducibility was performed utilizing root‐mean‐square standard deviation (SD_RMS_), a technique analogous to that widely employed in bone mineral densitometry reproducibility assessment.^17^, ^18^ Formulae used to calculate SD_RMS_ and LSC are presented in the Appendix. Results are presented as mean ± standard deviation.

## 3 RESULTS

**Figure 1:**
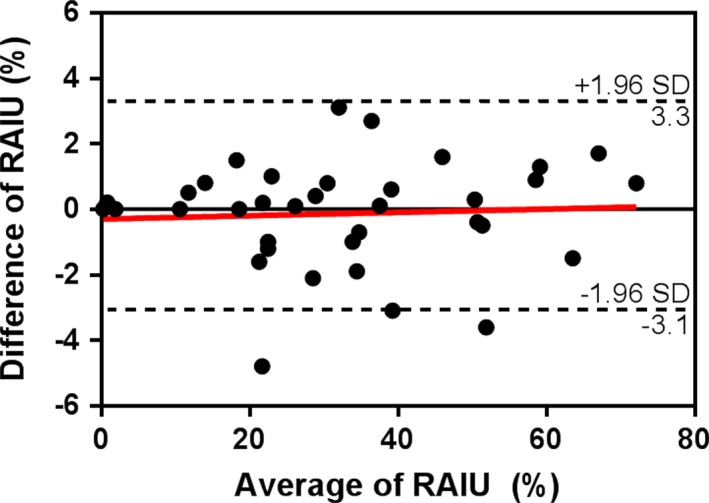
Bland–Altman plot showing the reproducibility of duplicated radioactive iodine uptake (RAIU) measurements in 36 patients. Mean difference and 95% confidence interval of mean of difference is indicated by the solid and dotted lines respectively. Linear correlation of difference versus average of the duplicated RAIU value is indicated by the red line.

**Figure 2:**
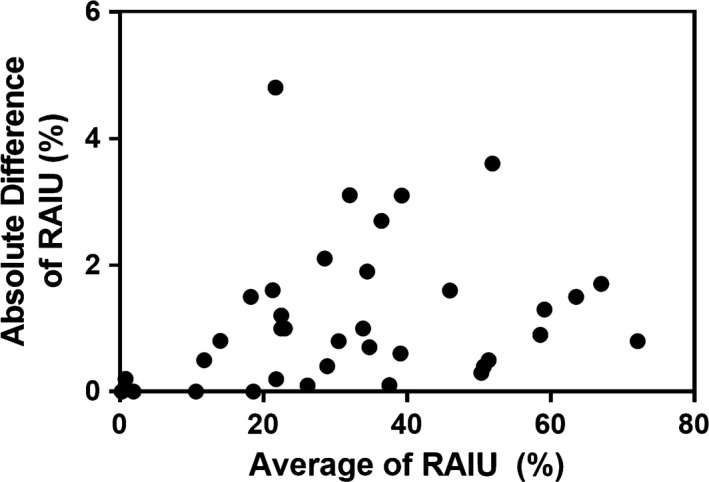
Absolute value of difference versus average of duplicated radioactive iodine uptake (RAIU) measurements in 36 patients.

Final diagnoses of the 36 patients included in the reproducibility analysis are listed in Table 1. Initial RAIU ranged from 0.3% to 72% (33 ± 19%) and second RAIU from 0.3% to 73% (33 ± 19%). Differences in RAIU between the repeated measurements ranged from −4.8% to 3.1% (−0.1 ± 1.6%) (Figs. 1 and 2) and the difference between test and retest value was not statistically significant (*P* = 0.63). There was no significant correlation between ΔRAIU and average RAIU (r = −0.06, *P* = 0.74). Reproducibility of the RAIU measurement was calculated to be 1.1% and absolute 95% LSC was 3.2%.

**Table 1:** Diagnoses of the 36 patients with duplicated measurements included in the reproducibility analysis

Final diagnosis	# patients
Thyroid cancer	2
Thyroiditis	3
Normal	4
Hyperthyroidism secondary to GD or toxic MNG	27
Total	36

GD, Graves’ disease; MNG, Multinodular goiter.

## 4 DISCUSSION

We have demonstrated a readily performed method to determine the reproducibility and LSC of RAIU in a typical hospital clinic. RAIU, following current guidelines within our clinic, is a reproducible measurement with a reproducibility that remained fairly constant across a wide range of RAIU values (0%–70%). Reproducibility of the measurement of RAIU was calculated to be 1.1%, yielding a 95% LSC of 3.2%. This degree of reproducibility exceeds the requisite 10% accuracy requirements of the dose calibrator,^20^ accuracy of thyroid size measurement, and variation in the quoted prescribed activity concentration K, all of which, like RAIU, directly affect magnitude of the calculated radioactive iodine dosage. The 1.1% reproducibility of RAIU measurement determined in our clinic is therefore not a limiting factor in the reproducibility of RAIT dosage prescription. In addition, our measurements confirm that variations in RAIU seen over time are mainly driven by physiologic and pathologic processes, with only a small contribution due to actual imprecision of the measurement method. The small variation observed between repeat RAIU measurements is likely related to variations in probe positioning and background as well as statistical fluctuations. It is unlikely that true variation in thyroid iodine concentrations contributed significantly to change in RAIU given that repeated measurements were obtained within several minutes of each other at 24‐h postingestion; fluctuations in count‐rate sensitivity should likewise be negligible over this short time interval. Variation in measurement highlights the importance of geometry, as variation in geometrical setup has a substantial effect on quantification. This study did not assess variation in absorption of the iodine pill.

Quantitative measurement is a distinguishing and appealing feature of nuclear medicine procedures. Nonetheless, reproducibility assessment is not routinely performed for several quantitative nuclear medicine procedures such as renal glomerular filtration rate, assessment of left ventricular ejection fraction with myocardial perfusion imaging, and dosimetry. In fact, as far as we can ascertain, this study is the first to formally assess clinical reproducibility of RAIU measurements. RAIU measurements are used to calculate the dosage of radioactive iodine to administer a patient. Because the dosage is inversely proportional to the RAIU measurement, if we wish to understand the tolerance of the predicted therapy activity, we need to understand the built‐in uncertainty in our estimate based on the reproducibility of RAIU. As well, knowledge of the reproducibility allows for better assessment of variation in calculated radiation exposure to the public and family members of patients treated with radioactive iodine. Finally, a prerequisite to understanding whether the RAIU value has changed over time to a statistically significant degree is a basic understanding of the reproducibility of the measurement. In this report, we have demonstrated the feasibility of obtaining reproducibility measurements for RAIU in a routine clinical laboratory. Assessment of reproducibility can be incorporated into a quality assurance program, and can be compared to prior historical or peer‐based values for comparison.

The main limitation of this study as a method of demonstrating the feasibility of measuring reproducibility, is that it relies on only a single model of thyroid probe device used at a single institution. It might be beneficial to illustrate this technique at different institutions, using different equipment, isotope of iodine, staff, patient population, and protocols. Nonetheless, this paper represents a first attempt to describe the technique for obtaining reproducibility estimates. This highlights the importance of each institution measuring their practice‐specific values. Significant variation from norms should prompt a review of equipment and technique including radioiodine dosage, probe characteristics, and technologists’ expertise.

## CONFLICT OF INTEREST

None.

## APPENDIX 1

### 1.1

Root‐mean‐square standard deviation (SD_RMS_) is computed using the following formula:

(2) $$SD_{\mathrm{RMS}}=\sqrt{\frac{\sum_{i=1}^{m}SD_{i}}{m}}$$

where SD_*i*_ represents the standard deviation of the repeated measures of the ith subject and *m* is the number of subjects. The least significant change (LSC), with one baseline and one follow up measurements, is computed according to the following formula:

(3) $$\mathrm{LSC}=Z^{\prime }\cdot \sqrt{2}\cdot SD_{\mathrm{RMS}}$$

where *Z*’ is a value chosen according to the level of statistical confidence. For statistical confidence levels of 80%, 90%, 95%, and 99%, the *Z*’ values are 1.28, 1.65, 1.96, and 2.58 respectively. A total number of 30 subjects is recommended when reproducibility is assessed with one baseline and one follow‐up measurement.^18^ Using 30 subjects with duplicated measurements provides 30 degrees of freedom, which ensure statistical validity of the analysis.^18^
